# In Vitro and In Vivo Studies of Anti-Lung Cancer Activity of *Artemesia judaica* L. Crude Extract Combined with LC-MS/MS Metabolic Profiling, Docking Simulation and HPLC-DAD Quantification

**DOI:** 10.3390/antiox11010017

**Published:** 2021-12-22

**Authors:** Marwa S. Goda, Mohamed S. Nafie, Basma M. Awad, Maged S. Abdel-Kader, Amany K. Ibrahim, Jihan M. Badr, Enas E. Eltamany

**Affiliations:** 1 Department of Pharmacognosy, Faculty of Pharmacy, Suez Canal University, Ismailia 41522, Egypt; marwa_saeed@pharm.suez.edu.eg (M.S.G.); amany_mohamed@pharm.suez.edu.eg (A.K.I.); gehan_ibrahim@pharm.suez.edu.eg (J.M.B.); Enastamany@gmail.com (E.E.E.); 2 Department of Chemistry, Faculty of Science, Suez Canal University, Ismailia 41522, Egypt; mohamed_nafie@science.suez.edu.eg; 3 Department of Pharmacognosy, Faculty of Pharmacy and Pharmaceutical Industries, Sinai University, El-Arish 45518, Egypt; basma.awad@su.edu.eg; 4 Department of Pharmacognosy, College of Pharmacy, Prince Sattam Bin Abdulaziz University 173, Al-Kharj 11942, Saudi Arabia; 5 Department of Pharmacognosy, College of Pharmacy, Alexandria University, Alexandria 21215, Egypt

**Keywords:** *Artemisia judaica*, A549 cell line, apoptosis, xenograft model, LC-MS/MS, CDK-2, EGFR, HPLC-DAD

## Abstract

*Artemisia judaica* L. (Family: Asteraceae) exhibited antioxidant, anti-inflammatory, and antiapoptotic effects. The in vitro cytotoxic activity of *A. judaica* ethanolic extract was screened against a panel of cancer cell lines. The results revealed its cytotoxic activity against a lung cancer (A549) cell line with a promising IC_50_ of 14.2 μg/mL compared to doxorubicin as a standard. This was confirmed through the downregulation of antiapoptotic genes, the upregulation of proapoptotic genes, and the cell cycle arrest at the G2/M phase. Further in vivo study showed that a solid tumor mass was significantly reduced, with a tumor inhibition ratio of 54% relative to doxorubicin therapy in a Xenograft model. From a chemical point of view, various classes of natural products have been identified by liquid chromatography combined with tandem mass spectrometry (LC-MS/MS). The docking study of the detected metabolites approved their cytotoxic activity through their virtual binding affinity towards the cyclin-dependent kinase 2 (CDK-2) and epidermal growth factor receptor (EGFR) active sites. Finally, *A. judaica* is a fruitful source of polyphenols that are well-known for their antioxidant and cytotoxic activities. As such, the previously reported polyphenols with anti-lung cancer activity were quantified by high-performance liquid chromatography coupled with a diode array detector (HPLC-DAD). Rutin, quercetin, kaempferol, and apigenin were detected at concentrations of 6 mg/gm, 0.4 mg/gm, 0.36 mg/gm, and 3.9 mg/gm of plant dry extract, respectively. It is worth noting that kaempferol and rutin are reported for the first time. Herein, *A. judaica* L. may serve as an adjuvant therapy or a promising source of leading structures in drug discovery for lung cancer treatment.

## 1. Introduction

Up-to-date information about global health care has revealed that cancer is considered to be the most predominant diseases worldwide, with a high mortality rate that led to 9.6 million deaths in 2020. As such, there is still a pressing need to develop novel effective and selective chemotherapeutic anticancer agents [1]. Lung cancer is one of the most aggressive and prominent causes of cancer-related death in both males and females. According to the World Health Organization (WHO), it is the dominant form of cancer in men, ranking first in terms of both incidence and mortality, while it ranks third in women in terms of incidence and second in terms of mortality [2]. Lung cancer can be classified into two subtypes: small lung cell carcinoma (SCLC) and non-small-cell lung carcinoma (NSCLC), representing nearly 80% of the lung cancer cases. Patients with advanced NSCLC face a poor prognosis, even when being treated under certain combination regimes [3]. Hence, it is important to investigate new treatments that may enhance the outcome of NSCLC patients [4]. Globally, herbal products have been used as health supplements or therapeutic agents in the treatment of diseases. Herbal products can enhance the actions and diminish the toxicity of conventional chemotherapeutic drugs [1]. *Artemisia judaica* L. (Arabic name, Shih Balady) belongs to the family Asteraceae and grows widely in the Mediterranean region [5]. *A.* *judaica* L. is widely used by Bedouins as an anthelmintic and antiseptic oil. It is also recommended for the treatment of skin disorders, weak immune systems, digestive system disorders, diabetes, inflammatory-related diseases, fungal infections, and arthritis [6,7,8]. Phytochemical investigations of *A. judaica* L. have revealed the presence of different chemical classes, terpenes, polyphenols, flavonoids, and bitter principles [8,9,10,11]. Several scientific reports have handled the biological activities of the crude extract, fractions of different polarities, isolated active fractions, or pure compounds. The ethanolic crude extract of *A. judaica* L. has shown anti-blastocystis, antihyperglycemic, antihyperlipidemic, antioxidant, anti-inflammatory, and antiapoptotic effects [5,12]. Additionally, the hydro-methanolic extract of the aerial parts proved its hypoglycemic activity through the inhibition of *α*-glucosidase and significant scavenging activity for 2,2-diphenyl-1-picrylhydrazyl (DPPH) radicals [13,14]. Moreover, the cytotoxic activity of the alcoholic crude extract was previously reported against hepatocellular carcinoma (HepG2), breast cancer (MCF-7), and colon tumor (LoVo) cell lines. This was explained through its ability to inhibit cell proliferation, angiogenesis, and inflammation in addition to its a beneficial protective effect against doxorubicin-induced toxicity in mice [15,16,17,18,19]. Both the ethyl acetate and petroleum ether fractions exhibited remarkable renal protective activity in hyperlipidemic and hyperglycemic rats [20]. The essential oil fraction possesses anthelmintic, analgesic, anti-inflammatory, antipyretic, and antimicrobial effects [11,21]. Concerning the isolated pure compounds, piperitone and trans-ethyl cinnamate showed pronounced insecticidal and antilarval activity against *Spodoptera littoralis* [22]. A sesquiterpene lactone known as judaicin exhibited a cardio-tonic effect slightly better than digoxin [23]. Another compound that was later identified as cirsimaritin demonstrated antioxidant, anticancer, anti-inflammatory, antimicrobial, antagonistic, antidiabetic, neurological, cardiovascular, and hepatoprotective activities [24,25,26]. Both apigenin and quercetin displayed selective cytotoxic activity against many types of cancer cell lines with low or no toxicity to normal cells [27,28]. The current study was oriented to scrutinize the in vitro and in vivo antitumor effect of *A. judaica* L. against lung carcinoma especially, against the A549 cell line, which is the most commonly used human non-small cell lung cancer cell line for both basic research and drug discovery. It also focused on the identification of *A. judaica* phytochemicals through liquid chromatography combined with the tandem mass spectrometry (LC-MS/MS) technique followed by a brief illustration of their cytotoxic activity through a molecular docking study. Moreover, the estimation of polyphenols with reported cytotoxicity against lung cancer was handled through high-performance liquid chromatography coupled with diode array detector (HPLC-DAD). 

## 2. Materials and Methods

### 2.1. Plant Material and Extraction Process

The collection and extraction processes were performed as mentioned before [5]. The aerial part of *A. judaica* L., which is sold under the name of Shih Baladi, was purchased from Egyptian market and was taxonomically identified. An amount of 300 g of *A. judaica* L. was soaked and extracted with ethanol (1 L × 3) at ambient temperature. The combined ethanolic extracts were concentrated in vacuo to afford 23 g of *A. judaica* L crude extract. 

### 2.2. In Vitro Cytotoxic Activity

#### 2.2.1. Cell Culture and MTT Cytotoxic Assay

Different cancer cell lines, such as prostate (PC-3), breast (MDA-MB-231), ovarian (A2780), and lung (A549) cancer cells lines, were purchased from the National Cancer Institute, Cairo, Egypt. Then, they were maintained in Dulbecco’s Modified Eagle Medium (DMEM, Sigma-Aldrich, St. Louis, MO, USA) and supplemented with 2 mM L-glutamine (Lonza, Belgium), 10% fetal bovine serum (FBS, Sigma-Aldrich, St. Louis, MO, USA), and 1% penicillin-streptomycin (Lonza, Belgium). Cells were plated at a density of 5 × 10^3^ cells in triplicate in 96-well plates. After 48 h, the cells were treated with the ethanolic extract of *A. judaica* L. at concentrations of 0.1, 1, 10, and 100 µg/mL. Cell viability was assessed after 48 h using the MTT assay kit (Promega, New York, NY, USA) [29]. An amount of 20 μL of MTT dye, 3-(4,5-dimethylthiazol-2-yl)-2,5-diphenyl-2H-tetrazolium bromide, was transferred into the wells, and the plate was incubated for a period of three hours. The absorbance was measured at 570 nm using an ELISA microplate reader (BIO-RAD, model iMark, Tokyo, Japan). The viability was calculated relative to doxorubicin, and the half-maximal inhibitory concentration (IC_50_) values were determined using the GraphPad prism 7 [30,31]. 

#### 2.2.2. Annexin V/PI Staifning and Cell Cycle Analysis

The apoptosis rate was quantified using annexin V-FITC (BD Pharmingen, San Diego, CA, USA). Cells were seeded into 6-well culture plates (3–5 × 10^5^ cells/well) and were incubated overnight. Then, the A549 cells were treated with the ethanolic crude extract of *A. judaica* L. for 48 h. After centrifugation, the supernatants and cells were collected and rinsed with ice-cold phosphate-buffered solution (PBS). The next step was suspending the cells in 100 µL of an annexin binding buffer solution that consisted of 25 mM CaCl_2_, 1.4 M NaCl, and 0.1 M Hepes/NaOH, pH 7.4, followed by incubation with an annexin V-FITC solution (1:100) and propidium iodide (PI) at a concentration of 10 µg/mL in the dark for 30 min. The stained cells were then detected using a Cytoflex FACS machine. Data were analyzed using the cytExpert software [32,33]. 

#### 2.2.3. RT-PCR for the Apoptosis-Related Genes

For further investigation of the apoptotic pathway, the study handled the gene expression of P53, Bax, and Caspapses-3,8,9 as pro-apoptotic genes as well as Bcl-2 as an anti-apoptotic gene (Table 1). The A549 cell lines were treated with the ethanolic crude extract of *A. judaica* L. with a dose that was equal to the IC_50_ value. After an incubation period of 48 h, the routine work of RNA extraction, cDNA synthesis, and RT-PCR reaction was carried out. All reactions were performed for 35 cycles using the following temperature profiles: 95 °C for 5 min (initial denaturation); 95 °C for 15 min (denaturation); 55 °C for 30 min (annealing); and 72 °C for 30 min (extension). The cycle threshold (Ct) values were collected to calculate the relative gene expression in all of the samples by normalization to the β-actin housekeeping gene [34,35].

### 2.3. In Vivo Experiment (Xenograft Model)

#### 2.3.1. Animals

Forty male *Swiss albino* mice with a body weight range of 21–28 g were purchased from the National Cancer Institute, Cairo University, Egypt, and were maintained under a normal day/night cycle and in a hygienic environment. The mice were adapted to the study conditions for 10 days prior to experimentation. Basal diet and water were provided ad libitum. 

#### 2.3.2. Study Design

The mice were equally and randomly divided into four groups. The first group was a normal control group, while the remaining three groups were inoculated with A549 cells. The tumor cells (1 × 10^6^ tumor cells/mouse) were injected subcutaneously into the right thigh of the hind limb. After ten days of tumor cell inoculation, masses of A549 tumors began to appear, and the three inoculated groups were subsequently classified into a control group of A549 tumor cells without any treatment, a group inoculated with A549 cells and treated with the ethanolic crude extract of *A. judaica* L., and a group inoculated with A549 cells and treated with doxorubicin as a standard [36]. The third group was treated with a dose of 100 mg/Kg BW/IP/daily of *A. judaica* L. crude extract for seven days. At the end of the treatment, all animals from all of the different groups were sacrificed, and both the weight and volume of the solid tumor masses were measured. 

#### 2.3.3. Biochemical Investigation and Histopathological Examination

Blood samples were collected and centrifuged to determine the hepatic enzymes, alanine transaminase (ALT), and aspartate aminotransferase (AST) using commercial kits (Instrumentation Laboratory SpA, Inova dignostics, Milano, Italy). For microscopic histopathological examination, pieces of the liver tissues from the animals of the four different groups were kept in 10% formalin, dehydrated in graded alcohol, embedded in paraffin sections, and stained with hematoxylin-eosin stain.

### 2.4. LC/Triple-TOF-MS/MS Metabolomic Analysis

High-performance liquid chromatography combined with the triple time-of-flight tandem mass spectrometry (LC/Triple-TOF-MS/MS) method was conducted as previously mentioned in detail [37,38,39]. An amount of 75 mg of the ethanolic crude extract of *A. judaica* L. were dissolved in 1.5 mL of a mixture containing water: methanol: acetonitrile (50:25:25) that was then ultra-sonicated and then centrifuged at 10,000 rpm for 10 min. An amount of 50 µL of the supernatant solution was taken and then completed to 1000 µL with the above-mentioned solvent mixture to attain a final concentration of 2.5 µg/µL. An injection volume of 10 µL was inoculated in both the positive and negative modes. The LC/Triple-TOF-MS/MS analysis was assessed using an ExionLC system (AB Sciex, Framingham, MA, USA) with an autosampler system, an in-line filter disks pre-column (0.5 µm × 3.0 mm, Phenomenex, Torrance, CA, USA), and an X select HSS T3 column (2.5 µm, 2.1 × 150 mm, Waters Corporation, Milford, MA, USA) sustained at 40 °C. The mobile phase composed of 5 mM ammonium formate buffer in 1% methanol with the pH adjusted to 3.0 for positive mode or adjusted to 8.0 for negative mode. The mobile phase was gradually eluted by increasing the concentration of the acetonitrile within 20 min, followed by a plateau period of 4 min, and finally, a decrease in the acetonitrile concentration within 3, min with a constant flow rate of 0.3 mL/min. This compartment was connected to a Triple TOF™ 5600+ system (AB SCIEX, Concord, ON, Canada) to detect the MS/MS transitions of the analytes. The detected metabolites were recognized by means of their *m/z* and MS/MS transitions compared to those found in recorded databases. Moreover, the MZmine ID, retention time, adduct formula, and molecular formula were detected. 

### 2.5. Molecular Docking Simulations of the Metabolites Detected by LC/Triple-TOF-MS/MS Analysis

The molecular docking study was conducted to determine the amounts of the cyclin-dependent kinase-2 (CDK-2) and epidermal growth factor receptor (EGFR) active sites. Proteins, (PDB = 2a4l) and (PDB: 1M17), were freely accessible from the protein data bank, PDB. Their structures were optimized by adjusting the amino acids with missing atoms or alternative positions, and then ligand structures were built, optimized, and energetically favored using Maestro. A molecular docking study was performed following routine preparation work for the appropriate receptor and ligand formats, the determination of grid box dimensions box of 10 Å in the x, y, and z directions centered on the ligand, and finally docking with binding activities in terms of binding energies and ligand–receptor interactions [40]. Finally, the molecular docking calculations were validated through MOE 2019 (Molecular Operating Environment Chemical Computing Group, Montreal, QC, Canada), and Chimera-UCSF software was utilized as a visualized software to assess the target drug interactions.

### 2.6. HPLC-DAD Analysis

#### 2.6.1. Standard Compounds

At first, nine reference polyphenol standards, namely gallic acid (≥98%), catechins (≥98%), chlorogenic acid (≥98%), ellagic acid (≥98%), rutin (≥98%), hesperidin (≥98%), quercetin (≥98%), kaempferol (≥98%), and apigenin (≥98%), were purchased from Nawah Scientific, Egypt.

#### 2.6.2. Apparatus and Operating Conditions

High-performance liquid chromatography (HPLC) analysis was assessed using the Waters 2690 Alliance HPLC system (Milford, CT, USA) equipped with a Waters 996 photodiode array detector. The combined methanolic solution of the nine different standards was prepared, and an amount of 10 µL was injected into the C18 column Inertsil ODS, which had the dimensions of 4.6 × 250 mm and a particle size of 5 µm. The mobile phase consisted of 0.1% phosphoric acid in water: acetonitrile with a constant flow rate of 1 mL/min, and a pH of 3.5 [41]. The mobile phase consisted of 0.1% phosphoric acid in water: acetonitrile, with a constant flow rate of 1 mL/min and a pH of 3.5 [41]. Gradient elution was conducted using a start mixture of acidic water: acetonitrile (95:5, *v/v* for 5 min and then increasing a concentration of acetonitrile to 80% over 30 min followed by a plateau period of 25 min, and finally, the acetonitrile concentration was gradually decreased to 10%, which was then held isocratically for 5 min. The absorbance was measured at 280 nm.

#### 2.6.3. Sample Preparation

An amount of 1 g of the ethanolic crude solution of *A. judaica* L. was accurately weighed, dissolved, sonicated for 15 min, filtered through a 0.22 µm Nylon syringe filter, and then an amount of 10 µL with a final concentration of 200 mg/mL was injected. 

#### 2.6.4. Calibration Graphs and Calculations

The stock methanolic solutions of the four selected reference standards, namely rutin, quercetin, kaempferol, and apigenin, were serially diluted to obtain different concentrations. Each one was filtered using a 0.22 µm syringe filter, and an amount of 10 µL was injected. The obtained peak areas were determined and plotted against the different concentrations. Finally, the slope of each trendline and the correlation coefficient were calculated.

## 3. Results and Discussion

### 3.1. In Vitro Cytotoxic Activity of the Ethanolic Crude Extract of A. judaica L.

#### 3.1.1. The Cytotoxic Activity of *A. judaica* L. Extract against A549 Cells Using MTT Assay

**Figure 1:**
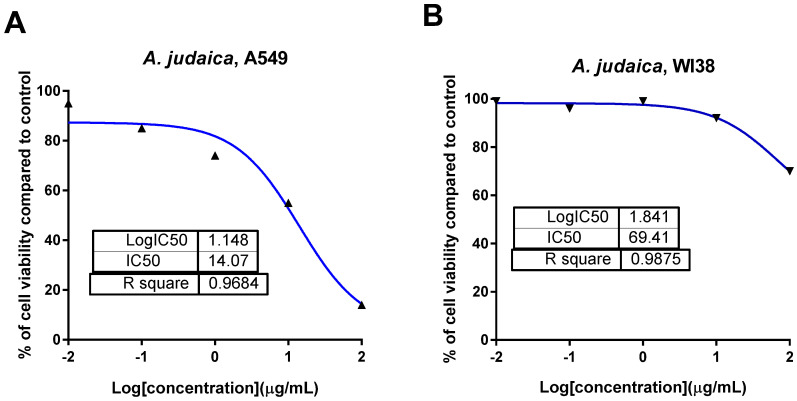
Dose–response nonlinear regression curve fitting the percentage of cell viability vs. log [con. µg/mL], R square ≈ 1 using the GraphPad prism software. (**A**): cytotoxicity against lung cancer A549 cells, and (**B**): cytotoxicity against normal lung WI38 cells.

The ethanolic extract of *A. judaica* L. was screened for its cytotoxicity against a panel of cancer cells, specifically PC-3, MDA-MB-231, A2780, and A549 cells, using the MTT assay, as seen in Table 2. The results exhibited that the crude extract showed potent cytotoxic activity against the A549 lung cancer cell line, with a promising IC_50_ of 14.2 μg/mL compared to doxorubicin as the standard drug (IC_50_ = 9.98 μg/mL).The crude extract exhibited minimal or low cytotoxic effect against the normal lung cells (WI38) in a selective way, with an IC_50_ value of 69.4 μg/mL (Figure 1). Our results agreed with a previous study that investigated the herbal plant *Artemisia* as a potential plant that could be used for cancer treatment [42].

#### 3.1.2. Effect of Crude Extract of *A. judaica* L. on Apoptosis Induction in A549 Cells Using Flow Cytometry

**Figure 2:**
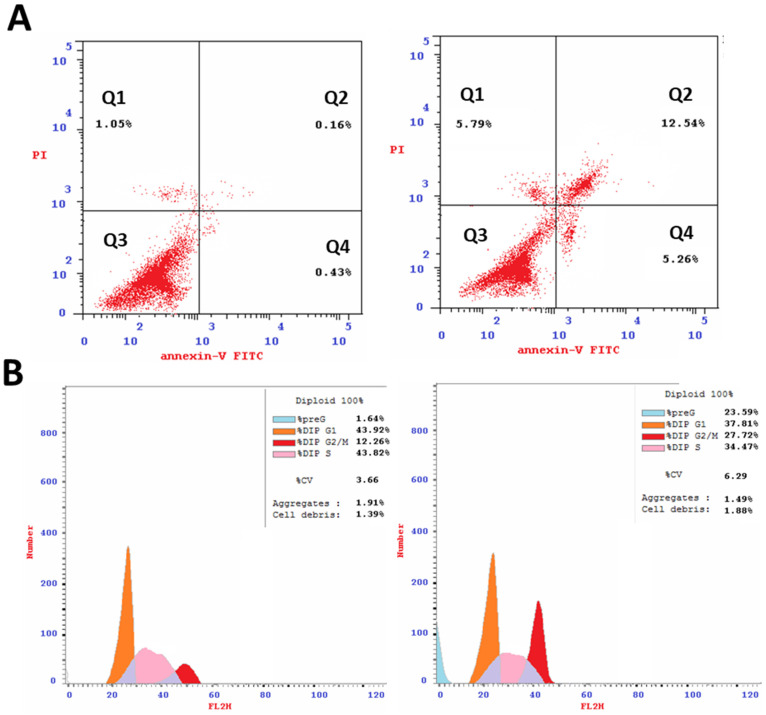
(**A**): FITC/annexin-V-FITC/PI differential apoptosis/necrosis of untreated and treated A549 cells with *A. judaica* L. ethanolic extract (IC_50_ of 14.07 µg/mL, 48 h). Quadrant charts show Q-1 (necrosis, AV–/PI+), Q-2 (late apoptotic cells, AV+/PI+), Q-3 (normal cells, AV–/PI–), and Q-4 (early apoptotic cells, AV+/PI–). (**B**): Cell cycle analysis of untreated and treated A549 cells.

Flow cytometric analysis of the annexin V/PI staining was performed to determine apoptotic cell death compared to necrotic cell death. The crude extract of *A. judaica* L. significantly stimulated apoptotic lung cancer cell death with 17.8% compared to 0.59% for the untreated control while it stimulated necrotic cancer cell death 5.79% compared to 1.05% (Figure 2A). As such, the crude extract of *A. judaica* L. favors the apoptotic cell death rather than the necrosis. After treatment with *A. judaica* L. ethanolic extract with a dose equal to its IC_50_ value, it was subjected to DNA flow cytometry to determine at which stage of cell cycle/cell proliferation was arrested. As seen in Figure 2B, the crude extract significantly increased the cell population at G2/M phase by 2.26-fold change (27.7% compared to control 12.26%). These results agreed with some of previous work which exhibited the apoptosis-inducing activity by arresting cell cycle at G2/M phase [19,43,44].

#### 3.1.3. Effect of Crude Extract of *A. judaica* L. on mRNA Gene Expression of Apoptosis-related Genes

**Figure 3:**
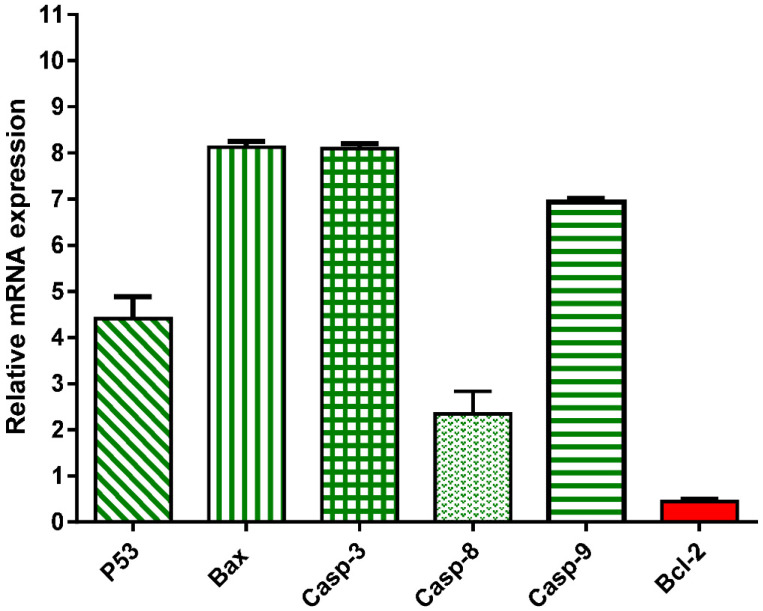
mRNA gene expression analysis of untreated and treated A549 cells with *A. judaica* ethanolic extract (IC_50_ of 14.07 µg/mL, 48 h). Fold of change of untreated control = 1.

To further validate the apoptosis-inducing activity of the crude extract of *A. judaica* L. (IC_50_ = 14.07 µg/mL, 48 h), the gene expression levels of pro-the apoptotic genes P53, Bax, and Caspase-3,8,9 and the anti-apoptotic genes Bcl-2 were investigated. As shown in Figure 3, the *A. judaica* L. crude extract increased the levels of P53, Bax, and caspases-3,8, and 9 by 4.41-fold, 8.1-fold, 8.09-fold, 2.3-fold, and 6.94-fold, respectively. On the other hand, it decreased the level of the antiapoptotic gene Bcl-2 level by 0.44-fold. As such, the crude extract of *A. judaica* L. was able to upregulate the proapoptotic genes and downregulate the antiapoptotic genes, proving the presence apoptotic cell death behavior. Our results agreed with previous studies that illustrated the apoptosis-inducing activity of upregulated Bax and cappase3 [16].

### 3.2. In Vivo Study (Xenograft Model)

#### 3.2.1. Effect of *A. judaica* Crude Extract on Tumor Mass Growth Xenografts

**Figure 4:**
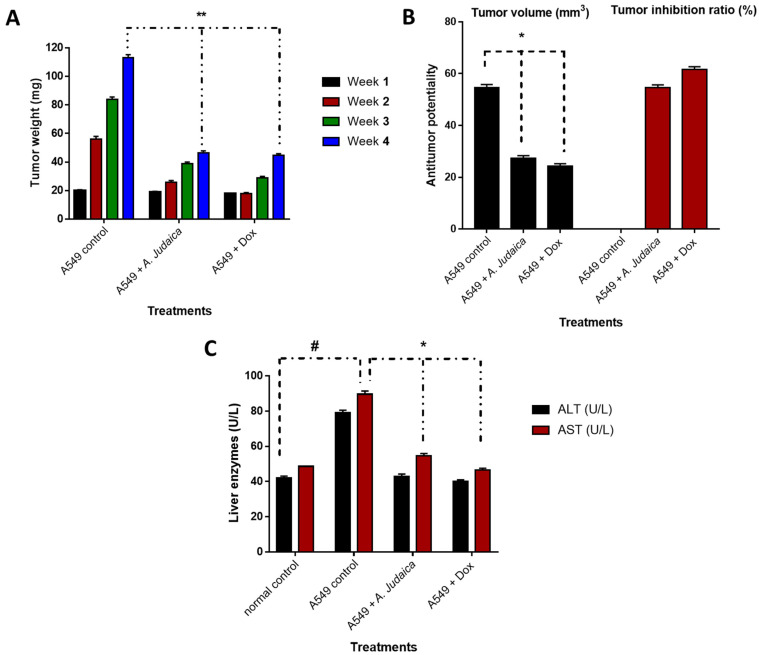
Anticancer activity of *A. judaica* L. and doxorubicin in the A549 group (Xenograft model) compared to in the control group. (**A**): Solid tumor mass (mg) during the experimental duration (4 weeks); (**B**): antitumor potentiality of tumor volume (mm^3^) and tumor inhibition ratio (TIR%); (**C**): biochemical measurements of liver enzymes (Alt and AST). Values are expressed as Mean ± SD values of mice in each group (*n* = 6). Sign (*) is significantly different (*p* ≤ 0.05), while sign (**) is highly significantly different (*p* ≤ 0.001) between the A549 control group and those treated using un-the paired test GraphPad prism. Sign (#) is significantly different (*p* ≤ 0.05) between the normal control group and the A549 control group.

To evaluate the anticancer activity of the crude extract of *A. judaica* L., both the tumor weight and tumor volume were observed. An increased solid tumor weight of about 113 mg was observed in a control A549 group over the experimental period. The antitumor activity of the crude extract of *A. judaica* L. as well as doxorubicin was interpreted as the solid tumor mass was significantly reduced to 46.5 mg and 44.62 mg, respectively, compared to the A549 control group. Additionally, the group that had been with the crude extract of *A. judaica* L. significantly inhibited the tumor volume, inhibiting the tumor volume by 54% (27.35 mm^3^) relative to doxorubicin therapy, where it demonstrated an inhibition rate of 61% (24.36 mm^3^) when compared to the A549 control group (54 mm^3^) (Figure 4).

#### 3.2.2. Biochemical Investigation and Histopathological Examination of Liver Tissues

**Figure 5:**
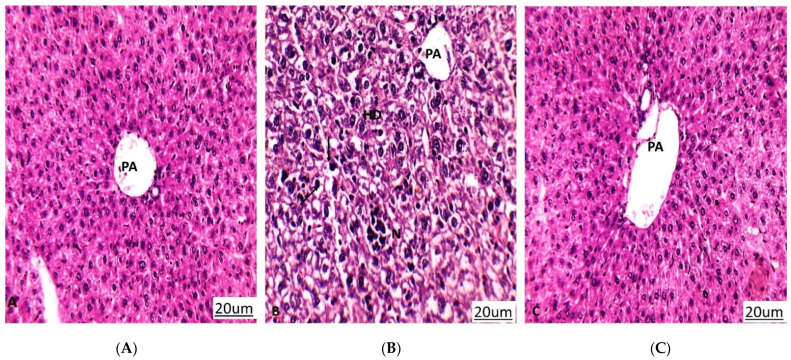
Histopathology of liver sections in differently treated groups. PA: portal area, HD: hydropic degeneration, N: necrosis, Black arrow: Pyknotic nucleus. (**A**) Normal control group that shows liver tissues with a normal architecture. (**B**) A549 group (untreated group) that shows hydropic degeneration of hepatocytes, loss of cell boundaries, pyknosis, and focal necrosis. (**C**) A549 group treated with *A. judaica* (100 mg/Kg BW) that shows improvement in liver tissues compared to the untreated A549 group. (Hematoxylin-eosin stain, magnification ×200).

Tumor proliferation inhibition and the amelioration of hepatic enzymes are the routine methods that are used to explain anticancer activity through in vivo models, examples of which have been previously published [45,46]. As a result of the hepatocellular damage that occurred after tumor inoculation, the ALT and AST hepatic enzymes were significantly elevated to be 79 and 89 (U/L), respectively, compared to 42 and 48 (U/L) in the normal mice. On the other hand, the hepatic enzymes were substantially reduced to be 43 and 54 U/L in the tumor-inoculated and normal mice, respectively, after treatment with the crude extract of *A. judaica* L., indicating a notable improvement in cancer-induced hepatocellular toxicity (Figure 5). In agreement with the improvement in hepatic enzymes, histopathological findings in the extracted liver tissues exhibited better improvement in the treated group than in the untreated group. The liver retained its normal hepatic architecture after treatment with the crude extract and was observed to be mostly normal with some minor degeneration.

### 3.3. LC/Triple-TOF-MS/MS Metabolomic Analysis of Ethanolic Crude Extract of Artemisia judaica L.

**Figure 6:**
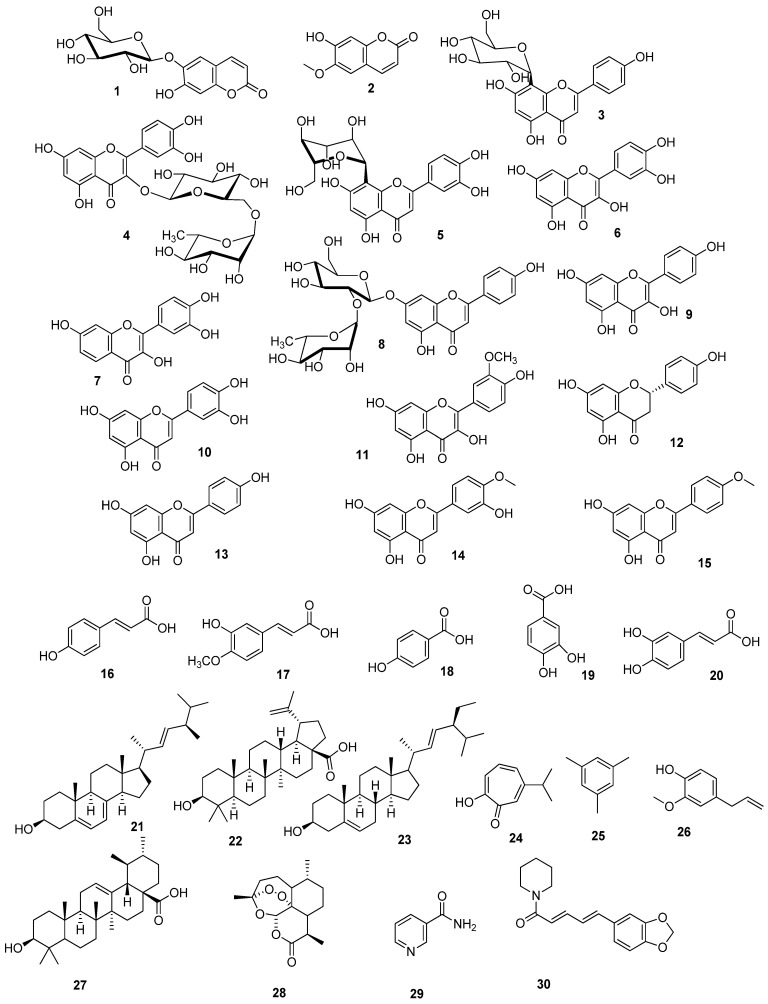
Chemical structures of the detected metabolites listed in Table 3.

Liquid chromatography combined with tandem mass spectrometry (LC-MS/MS) is a modern-day technique that is used for the detection of phytochemicals that may have a positive impact on human health. Herein, the study of the LC-MS/MS of the ethanolic crude extract of *Artemisia judaica* L. is handled for the first time. This metabolomic analysis manifested the presence of coumarins, flavonoids, flavonoid glycosides, phenolic acids, sterols, terpenes, terpenoid bitter principles, and alkaloids. These metabolites were deduced depending on their mass accuracy, which is expressed in parts per million (ppm) error [37,43,47], as well as matching their MS/MS ion transitions with those reported in literature (Table 3, Figure 6).

The multiple reaction monitoring (MRM) transition of coumarin compounds revealed the presence of esculin and scopoletin. Both esculin and scopoletin displayed antimicrobial activity against Gram-positive bacteria [71,72]. Additionally, scopoletin can regulate hyperglycemia through the regeneration of pancreatic *β*-cells and the attenuation of insulin resistance in high-fat diet/streptozotocin-induced diabetic mice [73]. Flavonoids and their glycosides are the most predominant class in *A. judaica* L. They are represented by quercetin, rutin, apigenin, rhoifolin, vitexin, kaempferol, fisetin, orientin, luteolin, isorhamnetin, naringenin, diosmetin, and acacetin. The therapeutic potential of these flavonoids has been described as antioxidant, neuroprotective, anti-mutagenic, anti-cancer, and anti-inflammatory agents [74,75,76,77,78,79,80,81,82,83,84]. Their anti-cancer activity is related to their ability to induce apoptosis and inhibit the angiogenesis process [74]. The antidiabetic activity of flavonoids was clarified by the regulation of carbohydrate metabolism and alleviating the sequential effects of hyperglycemia [85]. Regarding phenolic acids, the MS/MS ion transitions referred to *p*-coumaric acid, ferulic acid, *p*-hydroxy benzoic acid, protocatechuic acid, and caffeic acid. Phenolic acids are well known as potent antioxidants. Due to their antioxidant and anti-inflammatory activities, they have shown promising anticancer activity. Consequently, *P*-coumaric acid, ferulic acid, *P*-hydroxy benzoic acid, protocatechuic acid, and caffeic acid have been reported to have antioxidant, anti-inflammatory, anticarcinogenic, and hypoglycemic activities [86,87,88,89,90]. Obviously, *A. judaica* L. is plentiful in the polyhydroxylated phenolic compounds that may be responsible for the antioxidant, free radical scavenging, anti-inflammatory, antimicrobial, anti-angiogenic, and antihyperglycemic activities of the crude extract of *A. judaica* L. [12,13,22]. On the other hand, terpenes and terpenoid structures have been noticed. Terpenes comprise hinokitiol, mesitylene, eugenol, and ursolic acid, while terpenoid compounds include artemisinin, which possesses anthelmintic activity against round worms and potent anti-cancer activity [67,91] Moreover, both ursolic acid and eugenol have been shown to exhibit anti-inflammatory, antitumor, antiviral, and antimicrobial effects [92,93]. From literature, it was found that vitexin, orientin, quercetin, luteolin, apigenin, diosmetin, isoferulic acid, caffeic acid, santolina alcohol, mesitylene, and eugenol have been previously reported in *A. judaica* L. [8,11,13,94,95]. Betulinic acid was reported as an effective medicinal agent for the treatment of malignant melanoma [62], while alkaloid piperine has demonstrated anti-inflammatory and hypoglycemic effects [96]. 

The above-mentioned compounds play a beneficial role in the management of lung cancer (Table 4), which support the current in vitro and in vivo studies of the anti-lung cancer activity of the crude extract from *A. judaica* L. 

Finally, the current LC-MS/MS metabolomic analysis can be considered as a brief explanation for natural metabolites that may support the finds of the previously mentioned therapeutic activities of the crude extract from *A. judaica* L.

### 3.4. Molecular Docking Simulations

**Figure 7:**
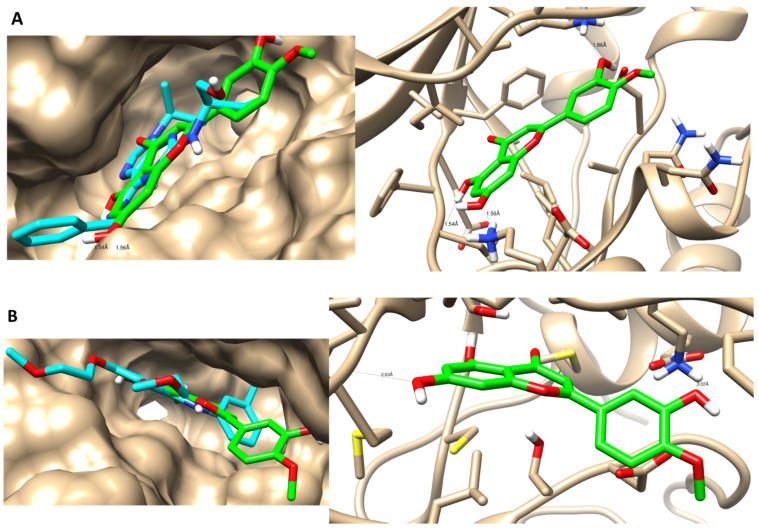
Binding disposition and ligand–receptor interactions of compound 14 inside the CDK-2 protein (**A**) and the EGFR protein (**B**). Left panel indicates surface representation, while the right panel indicates interactive mode. Three dimensional images were made using Chimera software.

The identified metabolites were screened for their binding affinity towards two proteins: cyclin-dependent kinase-2 (CDK-2) and epidermal growth factor receptor (EGFR), which represent two of the most common cell-signaling pathways that control cell survival and apoptosis [116]. The identified metabolites were docked inside the active sites of the proteins 2a4l and 1M17 with binding energies of (−9.58 to −19.89 Kcal/mol) and (−10.28 to −19.36 Kcal/mol), respectively. As seen in Table 5, the docking results illustrated that most of the docked metabolites formed a remarkable interaction binding mode with the two protein active sites, as they formed nearly the same interactions with the key amino acids with the co-crystallized ligand binds. Interestingly, metabolite 14 exhibited dual inhibition activity against CDK-2/EGFR, as it maintained the binding disposition in the same way that the co-crystallized ligand did and formed the same interactions with the key amino acids (Figure 7). Herein, the docking study proved the cytotoxic activity of the detected metabolites that may contribute to the cytotoxic activity of the crude extract of *A. judaica* L.

### 3.5. Identification and Quantification of Flavonoids by Using HPLC Analysis

#### 3.5.1. Qualitative Identification

**Figure 8:**
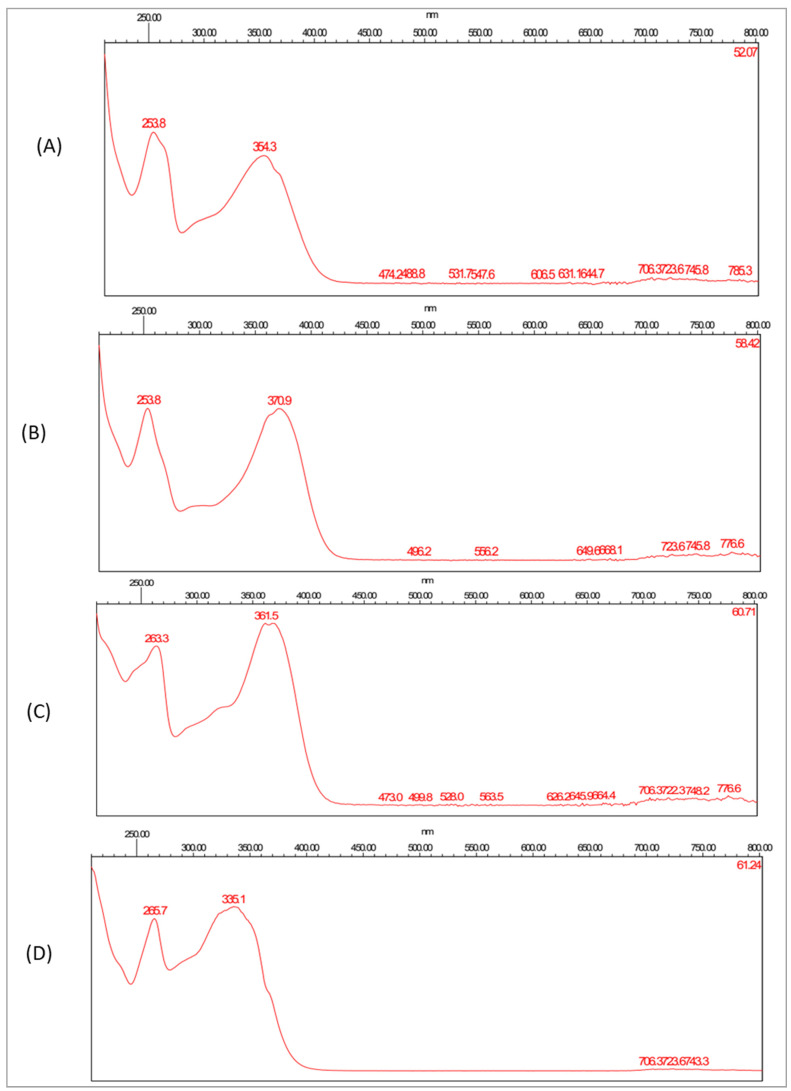
UV–vis absorbing spectrograms of rutin (**A**), quercetin (**B**), kaempferol (**C**), and apigenin (**D**).

**Figure 9:**
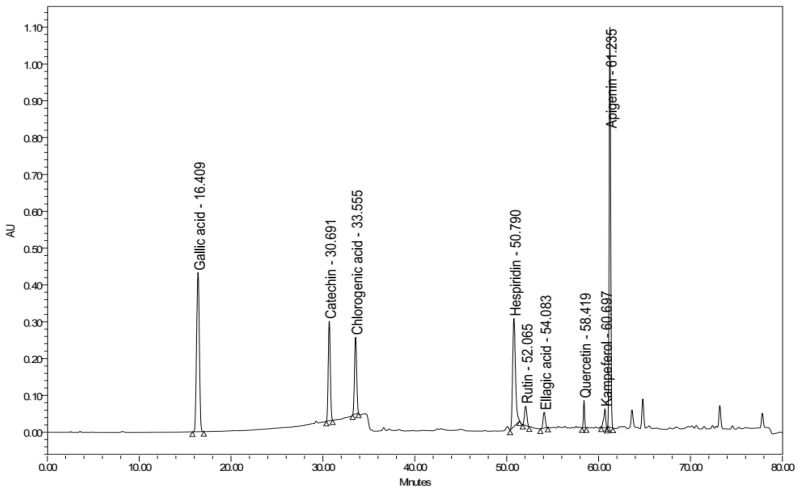
HPLC chromatogram of nine polyphenol reference standards at 280 nm.

**Figure 10:**
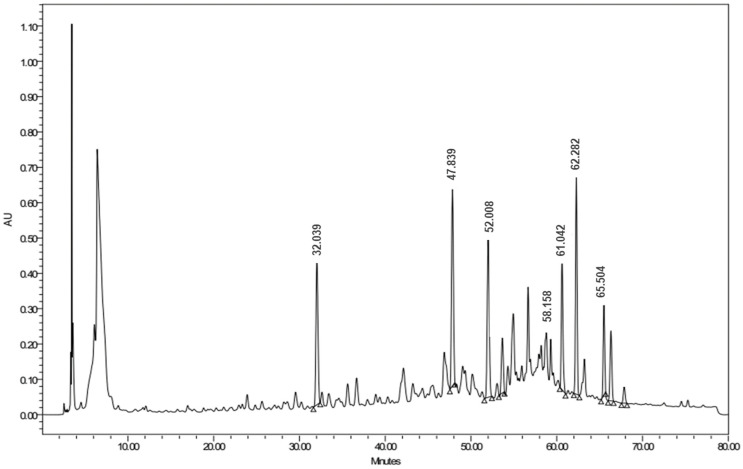
HPLC chromatogram of the ethanolic extract of *Artemesia judaica* L. (200 mg/mL) at 280 nm.

High-performance liquid chromatography combined with a diode array detector (HPLC-DAD) was established to identify the polyphenols that existed in the ethanolic extract of *A. judaica* L. by comparing both the retention time and UV spectra of different peaks that had been generated from the plant extract with those of nine different standards, namely gallic acid, catechin, chlorogenic acid, hesperidin, rutin, ellagic acid, quercetin, kaempferol, and apigenin (Figure 8). As such, the peaks that were eluted at 52, 58, 59, and 61 min could be identified as rutin, quercetin, kaempferol, and apigenin, respectively (Figure 9 and Figure 10). 

To the best of our knowledge, quercetin and apigenin were isolated earlier, but kaempferol and rutin are reported here for the first time. It is worth mentioning that the four identified flavonoids were previously reported to suppress the cell proliferation and to induce the apoptosis of A549 human lung carcinoma cells with minimal toxicity on normal cells [104,117,118,119]. Additionally, kaempferol is a potent cytotoxic agent that is able to act against different cancer cell lines, such as ovarian (A2780), lung (H460), skin (A431), pancreas (MIA PaCa-2), prostate (Du145), colon (HT29), and breast (MCF-7) cancer lines [120]. The same can be said for rutin, as it displayed anticancer effects toward a human renal cancer cell line (786-O) with minimal toxicity on the Vero kidney cells [121]. Likewise, apigenin displayed cytotoxic activity against many types of cancer cell lines, imparting low or no toxicity to the normal cells [28]. Quercetin exemplified cytotoxic activity against colon cancer cell lines (HT29 and HCT15), with a minimal effect on normal epithelial cells [27]. As such, further quantitative investigation of the above-mentioned flavonoids with previously reported cytotoxic activity against lung cancer was conducted. 

#### 3.5.2. Quantitative Estimation

Herein, the concentrations of the four identified flavonoids, known as rutin, quercetin, kaempferol, and apigenin, were determined through the use of external standard method. Therefore, the validation of the quantification method should be handled.

##### Linearity

The linearity of the HPLC method was assessed through analyzing five different concentrations of each standard, each in triplicate. A linear relationship was obtained over the concentration range in relation to the area. Both the correlation coefficient (R^2^) and linear regression equation for each standard were calculated and expressed (Table 6). 

##### System Precision

The system precision was confirmed through the determination of a certain concentration of a mixture of the standard solutions of the four flavonoids (50 µg/mL), which was applied in triplicate. The value of percent the relative standard deviation (%RSD) was calculated (Table 6). 

##### Method Precision 

The method precision was ensured through the injection of a certain concentration of the ethanolic extract from *A. judaica* L., which was repeated four time. The low %RSD value revealed the precision of the method, as shown in Table 6.

##### Limits of Detection and Quantification

Both the limit of detection and the limit of quantification parameters were calculated based on the following formulae: 3 σ/S and 10 σ/S, respectively, where σ is the standard deviation of the response, and S is the slope of the calibration curve (Table 6). 

##### Analytical Solution Stability 

To ensure the stability of the standard solutions, the analytical method was repeated under two different storage conditions, at 4 °C for 10 days as well as at ambient temperature for 2 days, and then compared to that of freshly prepared solutions. 

##### Sample Analysis 

The established method was conducted for the concurrent determination of rutin, quercetin, kaempferol, and apigenin in the *A. judaica* L., and the method was applied in triplicate. The concentration of the four different flavonoids was deduced based on the above-mentioned regression equations. Finally, the concentrations of rutin, quercetin, kaempferol, and apigenin were found to be 6 mg/gm, 0.41 mg/gm, 0.36 mg/gm, and 3.9 mg/gm of plant dry extract, respectively.

## 4. Conclusions

In conclusion, the present study introduces *Artemesia judaica* L. as a promising cytotoxic agent against lung carcinoma through the induction of cell apoptosis, the inhibition of cell proliferation, and cell cycle arrest. Moreover, it is considered to be a plentiful source of polyphenols that have demonstrated well-known and potent antioxidant activities that contribute to anticancer activity. 

## Figures and Tables

**Table 1:** List of sequences in forward and reverse of tested genes.

Gene	Forward	Reverse
P53	5′-CCCCTCCTGGCCCCTGTCATCTTC-3′	5′-GCAGCGCCTCACAACCTCCGTCAT-3′
BAX	5′-GTTTCATCCAGGATCGAGCAG-3′	5′-CATCTTCTTCCAGATGGTGA-3′
CASP-3	5′-TGGCCCTGAAATACGAAGTC-3′	5′-GGCAGTAGTCGACTCTGAAG-3′
CASP-8	5′-AATGTTGGAGGAAAGCAAT-3′	5′-CATAGTCGTTGATTATCTTCAGC-3′
CASP-9	5′-CGAACTAACAGGCAAGCAGC-3′	5′-ACCTCACCAAATCCTCCAGAAC-3′
BCL2	5′-CCTGTGGATGACTGAGTACC-3′	5′-GAGACAGCCAGGAGAAATCA-3′
β-actin	5′-GTGACATCCACACCCAGAGG-3′	5′-ACAGGATGTCAAAACTGCCC-3′

**Table 2:** Cytotoxic activity of ethanolic crude extract of *A. udaica* L. against prostate, breast, ovarian, and lung cancer cell lines using the MTT assay.

Sample	IC_50_ (μg/mL) *			
	Prostate PC-3	Breast MDA-MB-231	Ovarian A2780	Lung A549
*A. judaica* L. crude extract	59.8 ± 3.25	98.6 ± 4.65	NA	14.2 ± 0.84
Doxorubicin	9.36 ± 1.52	7.26 ± 0.98	2.36 ± 0.65	9.98 ± 0.97

* Values are expressed as mean ± SD of 3 independent trials (*n* = 3). NA = Not active. IC_50_ (μg/mL) values were calculated using GraphPad Prism 7 software.

**Table 3:** LC-MS/MS metabolomic analysis of the ethanolic crude extract of *A. judaica* L.

	Polarity Mode	MZmine ID	Ret. Time (min)	Measured *m*/*z*	Calculated *m*/*z*	Mass Error (ppm)	Adduct	Molecular Formula	MS/MS Spectrum	Deduced Compound	Ref
Coumarins and their glycosides											
1	Negative	801	4.07	339.0659	339.0716	−16.81	[M − H]^−^	C_15_H_16_O_9_	339.1 > 177	Esculin	[48]
2	Positive	1717	7.45	193.0517	193.0501	8.29	[M + H]^+^	C_10_H_8_O_4_	193 > 178 > 133 > 122	Scopoletin	[49]
Flavonoids and their glycosides											
3	Negative	1480	6.59	431.0977	431.0978	−0.23	[M − H]^−^	C_21_H_20_O_10_	431.1 > 311.1	Vitexin	[50]
4	Positive	1434	6.73	611.1583	611.1612	−4.75	[M + H]^+^	C_27_H_30_O_16_	611.1 > 303.1	Rutin	[51]
5	Negative	1631	6.92	447.0922	447.0927	−1.12	[M − H]^−^	C_21_H_20_O_11_	447.1 > 327.1	Orientin	[50]
6	Positive	1566	7.13	303.0507	303.0505	0.66	[M + H]^+^	C_15_H_10_O_7_	303 > 257 > 229 > 183 > 165 > 153 > 137	Quercetin	[52]
7	Positive	1579	7.16	287.0554	287.0556	−0.7	[M + H]^+^	C_15_H_10_O_6_	287 > 269 > 241 > 213 >149 >137	Fisetin	[53]
8	Negative	1793	7.42	577.1612	577.1557	9.53	[M − H]^−^	C_27_H_30_O_14_	577.1 > 269.1	Rhoifolin	[54]
9	Positive	1869	7.76	287.0525	287.0536	−3.83	[M + H]^+^	C_15_H_10_O_6_	287.2 > 231 > 165.1 > 121.0	Kaempferol	[53]
10	Negative	2065	8.59	285.0405	285.0399	2.10	[M − H]^−^	C_15_H_10_O_6_	285.2 > 133.0	Luteolin	[55]
11	Negative	2068	8.65	315.0505	315.0505	zero	[M − H]^−^	C_16_H_12_O_7_	315.1 > 300.1	Isorhamnetin	[56]
12	Negative	2171	9.23	271.0601	271.0606	−1.84	[M − H]^−^	C_15_H_12_O_5_	271 > 177 > 151	Naringenin	[49]
13	Negative	2324	10.08	269.0453	269.0450	1.12	[M − H]^−^	C_15_H_10_O_5_	269.2 > 116.8	Apigenin	[55]
14	Positive	2739	10.34	301.0680	301.0701	−6.98	[M + H]^+^	C_16_H_12_O_6_	301.1 > 286 > 258.0	Diosmetin	[57]
15	Positive	3059	11.52	285.0761	285.0763	−0.7	[M + H]^+^	C_16_H_12_O_5_	285.0 > 267.9 > 242.1	Acacetin	[58]
Phenolic acids											
16	Negative	590	1.62	163.0394	163.0395	−0.61	[M − H]^−^	C_9_H_8_O_4_	163 > 119	*p*-Coumaric acid	[59]
17	Negative	725	2.43	193.0514	193.0501	6.73	[M − H]^−^	C_10_H_10_O_4_	193 > 178> 149 > 134	Ferulic acid	[59]
18	Negative	751	3.30	137.0222	137.0231	−6.6	[M − H]^−^	C_7_H_6_O_3_	137 > 93 > 65	*p*-Hydroxy benzoic acid	[60]
19	Negative	991	4.97	153.0166	153.0178	−7.8	[M − H]^−^	C_7_H_6_O_4_	153 > 109	Protocatechuic acid	[60]
20	Negative	1163	5.55	179.0336	179.0344	−4.47	[M − H]^−^	C_9_H_8_O_4_	179 > 135 > 134	Caffeic acid	[60]
Sterols											
21	Positive	5376	21.9	380.3320	380.3343	−6.05	[M + H − H_2_0]^+^	C_28_H_44_O	380 > 69	Ergosterol	[61]
22	Negative	3245	22.84	455.3567	455.3525	9.22	[M − H]^−^	C_30_H_48_O_3_	455	Betulinic acid	[62]
23	Positive	5641	23.74	413.3615	413.3633	−4.35	[M + H]^+^	C_29_H_48_O	413 > 395.3 > 81.1	Stigmasterol	[63]
Terpenes											
24	Negative	1071	5.29	163.0754	163.0759	−3.07	[M − H]^−^	C_10_H_12_O_2_	163 > 146 > 119	Hinokitiol/*β*-thujaplicin	[64]
25	Positive	2327	8.83	121.1004	121.1017	−10.73	[M + H]^+^	C_9_H_12_	121 > 119 > 105 > 91 > 77	Mesitylene	[65]
26	Positive	3393	12.85	165.0897	165.0916	−11.5	[M + H]^+^	C_10_H_12_O_2_	165 > 149 > 103	Eugenol	[66]
27	Negative	3245	22.84	455.3567	455.3525	9.22	[M − H]^−^	C_30_H_48_O_3_	455	Ursolic acid	[67]
Terpenoid bitter principles											
28	Positive	5246	21.18	283.1499	283.1545	−16.25	[M + H]^+^	C_15_H_22_O_5_	283 > 265 > 247 > 237 > 209	Artemisinin	[68]
Other classes											
29	Positive	64	1.22	123.0553	123.0558	−4.06	[M + H]^+^	C_6_H_6_N_2_O	123 > 80	Nicotinamide	[69]
30	Positive	3829	15.21	286.1450	286.1443	2.45	[M + H]^+^	C_17_H_19_NO_3_	286.1 > 201 > 171 > 143 > 135	Piperine	[70]

**Table 4:** The anti-lung cancer activity of some detected metabolites.

No.	Detected Metabolite	Type of Study	Mechanism of Action	Ref.
1	Vitexin	-Animal study with acute lung injury	Vitexin upregulated nuclear factor erythroid-2-related factor2 (Nrf2) and activated heme oxygenase (HO)-1.	[81]
2	Rutin	-Human lung cancer cell line, A549	Rutin downregulated the expression of anti-apoptotic gene (Bcl-2) and decreased the levels of tumor necrosis factor (TNF-*α*).	[97]
3	Quercetin	-Human lung cancer cell line, A549 -Xenograft animal model	Quercetin downregulated the expression of anti-apoptotic gene (Bcl-2) and upregulated the expression of the proapoptotic gene (Bax).	[98]
4	Fisetin	-Human lung cancer cell line, A549	Fisetin induced cell cycle arrest at G2 phase and upregulated the expression of the apoptosis-regulating gene (Caspases 3 and 9).	[99]
5	Kaempeferol	-Human lung cancer cell line, A549	Kaempeferol upregulated the expression of proapoptotic gene (Bax) and downregulated the expression of anti-apoptotic genes (Bcl-2 and Bcl-xL).	[100]
6	Luteolin	-Human lung cancer cell line, A549	Luteolin suppressed migration and invasion of lung cancer cells.	[101]
7	Isorhamnetin	-Human lung cancer cell line, A549 -Animal model with Lewis lung cancer cells	Isorhamnetin upregulated the expression of proapoptotic genes (Bax, P53 and Caspase-3) and downregulated the expression of anti-apoptotic genes (Bcl-2, cyclinD1).	[102]
8	Naringenin	-Human lung cancer cell line, A549	Naringenin suppressed migration of lung cancer cells, upregulated the expression of proapoptotic genes (Bax and Caspase-3) and downregulated the expression of matrixmetallo proteinases (MMP-2 and MMP-9).	[103]
9	Apigenin	-Human lung cancer cell line, A549	Apigenin suppressed migration of lung cancer cells and downregulated the expression of MMP-9.	[104]
10	Diosmetin	-Human lung cancer cell lines, A549, H1299, H460, SPC-A1, H441, H1650 and Calu-3. -Xenograft animal model	Diosmetin induced apoptosis and enhanced the efficacy of paclitaxel, a chemotherapeutic agent.	[105]
11	Acacetin	-Human lung cancer cell line, A549	Acacetin suppressed migration of lung cancer cells and downregulated the expression of MMP-2 and 9.	[106]
12	Ferulic acid	-Human lung cancer cell line, H1299	Ferulic acid suppressed migration of lung cancer cells and downregulated the expression of MMP-2 and 9.	[107]
13	*p*-Hydroxy benzoic acid	-Human lung cancer cell line, A549	*p*-Hydroxy benzoic acid upregulated the expression of proapoptotic gene (Caspase-1) and interleukines (IL1β, and IL18).	[108]
14	Protocatechuic acid	-Human lung cancer cell lines, A549, H3255, and Calu-6	Protocatechuic acid upregulated the expression of proapoptotic genes (Bax and Caspase-3) and downregulated the expression of anti-apoptotic genes (Bcl-2) and matrixmetallo proteinases.	[109]
15	Caffeic acid	-Human lung cancer cell line, H1299 -Xenograft animal model	Caffeic acid enhanced the efficacy of paclitaxel and upregulated the expression of Caspases-3 and 9.	[110]
16	Ergosterol	-Human lung cancer cell line, A549	Ergosterol suppressed the proliferation of lung cancer cells.	[111]
17	Eugenol	-Human lung cancer cell line, A549 -Human embryonic lung fibroblast, MRC-5	Eugenol suppressed migration of lung cancer cells and downregulated the expression of MMP-2.	[112]
18	Hinokitiol	-Human lung cancer cell line, A549	Hinokitiol suppressed migration of lung cancer cells, downregulated the expression of MMP-2 and upregulated the expression of proapoptotic genes (Bax, P53 and Caspase-3).	[113]
19	Ursolic acid	-Human lung cancer cell line, A549	Ursolic acid suppressed migration of lung cancer cells and downregulated miR-21 that is correlated with a tumor growth.	[114]
20	Piperine	-Human fibrosarcoma cell, HT-1080 -B16F10 melanoma animal model	Piperine suppressed metastasise and migration of lung cancer cells.	[115]

**Table 5:** Summary of ligand–receptor interactions of the identified docked compounds towards cyclin-dependent kinase (CDK2) and Epidermal growth factor receptor (EGFR) binding sites. * *p* ≤ 0.05; ^#^, *p* ≤ 0.05.

Co-Crystallized Ligands (Key Interactions)	Ligand-Receptor Interactions towards CDK-2 (PD’a4l) *	Ligand-Receptor Interactions towards EGFR (PDB = 1M17) ^#^
	2 HB with Leu 83 + 1 Arene-Cation Interaction with Lys 89	1 HB with Met 769
Metabolite 1	1 HB with Lys 89	1 HB with 769
Metabolite 2	-	-
Metabolite 3	1 HB with Lys 89	-
Metabolite 4	1 HB with Lys 89	-
Metabolite 5	1 HB with Lys 89 + 1 arene-cation interaction with Lys 89	1 HB with 769
Metabolite 6	1 HB with Leu 83	1 HB with 769
Metabolite 7	1 HB with Lys 89 + 1 arene-cation interaction with Lys 89	1 HB with 769
Metabolite 8	1 arene-cation interaction with Lys 89	-
Metabolite 9	1 HB with Leu 83 + 1 arene-cation interaction with Lys 89	-
Metabolite 10	1 HB with Leu 83	1 HB with 769
Metabolite 11	1 HB with Leu 83	-
Metabolite 12	1 HB with Lys 89	1 HB with 769
Metabolite 13	-	-
Metabolite 14	2 HB with Leu 83 and Lys 89	1 HB with Met 769
Metabolite 15	1 arene-cation interaction with Lys 89	-
Metabolite 16	-	-
Metabolite 17	-	-
Metabolite 18	-	-
Metabolite 19	-	
Metabolite 20	1 arene-cation interaction with Lys 89	1 HB with 769
Metabolite 21		
Metabolite 22	1 HB with Lys 89	-
Metabolite 23	1 HB with Leu 83	
Metabolite 24	-	-
Metabolite 25	-	-
Metabolite 26	-	-
Metabolite 27	1 HB with Leu 83 + 1 arene-cation interaction with Lys 89	1 HB with Met 769
Metabolite 28	-	-
Metabolite 29	-	-
Metabolite 30	1 HB with Leu 83	-

**Table 6:** Validation parameters of the HPLC method for the simultaneous quantification of rutin, quercetin, kaempferol, and apigenin scanned at λ = 280 nm.

Validation Parameters	Rutin	Quercetin	Kaempferol	Apigenin
Regression equation	y = 13,199x − 787,148	y = 15,764.37x − 40,216.27	y = 71,227x − 46,571	y = 8938.8x + 357,854
Correlation coefficient (R^2^)	0.997	0.999	0.998	0.993
Linearity range (µg/mL)	10–200	5–100	10–150	5–100
Limit of detection (µg/mL)	0.7	0.5	0.4	0.5
Limit of quantification (µg/mL)	2.5	1.61	1.4	1.53
System precision (%RSD)	3.16	2.25	1.59	2.26
Method precision (%RSD)	1.93	2.78	2.19	1.31
Concentration (mg/gm)	6 ±0.019	0.413 ± 0.00007	0.3603 ± 0.0033	3.9 ± 0.007

RSD: relative standard deviation.

## Data Availability

Data is available within the article.
